# Dynamics of Protein Phosphatase Gene Expression in *Corbicula fluminea* Exposed to Microcystin-LR and to Toxic *Microcystis aeruginosa* Cells

**DOI:** 10.3390/ijms12129172

**Published:** 2011-12-08

**Authors:** José Carlos Martins, João Machado, António Martins, Joana Azevedo, Luís OlivaTeles, Vitor Vasconcelos

**Affiliations:** 1 CIIMAR/CIMAR, Interdisciplinary Centre of Marine and Environmental Research, University of Porto, Rua dos Bragas 289, 4050-123 Porto, Portugal; E-Mails: jcmmartins@hotmail.com (J.C.M.); jprmachado@gmail.com (J.M.); amartinstz@net.sapo.pt (A.M.); joana_passo@hotmail.com (J.A.) loteles@fc.up.pt (L.O.T.); 2 Department of Biology, Faculty of Sciences, University of Porto, Rua do Campo Alegre, 4069-007 Porto, Portugal

**Keywords:** *C. fluminea*, cyanotoxins, gene expression, microcystin-LR, protein phosphatases

## Abstract

This study investigated the *in vivo* effects of microcystins on gene expression of several phosphoprotein phosphatases (PPP) in the freshwater clam *Corbicula fluminea* with two different exposure scenarios. Clams were exposed for 96 h to 5 μg L^−1^ of dissolved microcystin-LR and the relative changes of gene expression of three different types of PPP (PPP1, 2 and 4) were analyzed by quantitative real-time PCR. The results showed a significant induction of PPP2 gene expression in the visceral mass. In contrast, the cyanotoxin did not cause any significant changes on PPP1 and PPP4 gene expression. Based on these results, we studied alterations in transcriptional patterns in parallel with enzymatic activity of *C. fluminea* for PPP2, induced by a *Microcystis aeruginosa* toxic strain (1 × 10^5^ cells cm^−3^) during 96 h. The relative changes of gene expression and enzyme activity in visceral mass were analyzed by quantitative real-time PCR and colorimetric assays respectively. The clams exhibited a significant reduction of PPP2 activity with a concomitant enhancement of gene expression. Considering all the results we can conclude that the exposure to an ecologically relevant concentration of pure or intracellular microcystins (-LR) promoted an *in vivo* effect on PPP2 gene expression in *C. fluminea*.

## 1. Introduction

Cyanobacterial blooms often produce toxic metabolites, including cyclic hepatotoxins microcystins (MC). Among more than 80 MC identified, MC-LR is one of the most toxic and commonly detected MC congeners in natural blooms [1,2]. MC-LR enters in the aquatic food web by assimilation of aquatic invertebrates and can be passed to a higher trophic level through accumulation in tissues [3]. These cyclic heptapeptides are strong inhibitors of several serine/threonine (Ser/Thr) protein phosphatases (PP) leading to cell’s metabolism alteration [4]. The reversible phosphorylation of proteins in eukaryotes catalyzed by protein kinases and Ser/Thr PP determines the biological activities of many proteins and is recognized as a major mechanism controlling diverse cellular processes [5]. In mammals, this inhibition mechanism is directly related to MC hepatotoxicity and tumor promotion activity [6–11]. In contrast to mammals, relatively few studies have focused on the uptake and toxicity of MC to aquatic organisms. Although there has been some research examining the capacity of accumulation and depuration of MC [3] by these organisms, there is little information about detrimental effects of exposure to these toxins and their interaction with cell’s targets, in particular with Ser/Thr PP.

Eukaryotic Ser/Thr PP are structurally and functionally diverse enzymes that are classified into three distinct gene families. Phosphoprotein Phosphatases (PPP) is one of those families which are responsible for phosphoserine and phosphothreonine dephosphorylation [12]. The PPP family of Ser/Thr PP comprises seven enzymes (PPP1-7), in a total of 13 catalytic subunits [13]. Ser/Thr PP are closely related in amino acid sequence but can be distinguished functionally based on inhibition by small molecular toxins such as MC-LR [14]. Goldberg *et al*. [15] found that MC-LR interacts with three regions on the surface of the catalytic subunit of PPP1 α isoform; the hydrophobic moiety, *C*-terminal moiety and the acidic moiety. MC undergo a two-step interaction with Ser/Thr PP which consists in a rapid and reversible binding followed by a covalent bound after several hours [16]. MC-LR, -LA and -LL were found to interact with PPP2 and PPP1 catalytic subunits by a two-step mechanism involving rapid binding and inactivation of the PP catalytic subunit within minutes, followed by a slower covalent interaction during prolonged reaction-time [16]. The methyl-dehydroalanine (Mdha) residue of the toxin forms a covalent linkage with Cysteine-273 and 269 of PPP1 and PPP2 respectively [15–17]. In mammals, inhibition of Ser/Thr PPs by MC results in the reorganization of cytoskeleton components and disruption of hepatic architecture, leading to severe and irreversible damages and potentially death [18]. Severe effects of MC were also shown in aquatic organisms such as decrease in survival, growth and fecundity, histopathological damage and oxidative stress induction [19–24]. Among this group of organisms, bivalves may be one of the most threatened group of aquatic organisms in the presence of toxic cyanobacteria blooms. Bivalves are sedentary mollusks and filter large amounts of water to handle with nutritional and respiratory needs, being able to accumulate cyanobacterial toxins [3].

One long-standing pitfall in toxicology is to attribute a toxic effect from a sample to a particular compound, without checking that the pure compound actually has that effect, leading to misleading conclusions [25]. *Microcystis* extracts and toxic cultures have been widely used in oral toxicological studies of MC as effective replicates of natural poisoning for several organisms by toxic cyanobacteria [26]. However, validation of the results with pure MC is in most cases neglected. In this study, we aimed to investigate the *in vivo* effects of dissolved MC-LR and *M. aeruginosa* toxic strain cells on gene expression of PPP in the freshwater clam *C. fluminea*. The assessment of mRNA levels has been used as a reliable method to measure xenobiotic or natural toxins stress [18,27]. We started by investigating several PPP from *C. fluminea*, identifying PPP1, 2 and 4 transcripts in the freshwater bivalve. Then, the gene expression of PPP1, 2 and 4 in the visceral mass of *C. fluminea* was evaluated in an exposure assay using purified MC-LR. Finally, we studied alterations in transcriptional patterns in parallel with enzymatic activity of *C. fluminea* for PPP2, following exposure to a strain of *M. aeruginosa* producing almost exclusively MC-LR.

## 2. Results and Discussion

In this work, we showed for the first time the presence of PPP1, PPP2 and PPP4 in *C. fluminea*. We amplified partial sequences of those genes in this species and the obtained sequences were deposited in GenBank with the accession numbers: JN379818; JN379819; JN379820. In mammals, the main functions of PPP1 are connected to muscle contraction/relaxation, glycogen metabolism, synaptic transmission, gene expression, RNA splicing and cell-cycle progression [13]. PPP2 and PPP4 catalytic subunits are phylogenetically related and have 65% amino acid sequence identity [28]. Although most closely related, PPP2 and PPP4 have individual functions and do not complement each other [29]. PPP2 is mainly cytosolic and plays a role in cell-cycle regulation, cell growth control, cytoskeleton dynamics, cell mobility, metabolism, transcription, translation, RNA splicing, DNA replication, apoptosis, inflammation and differentiation [13]. PPP4 is predominantly nuclear where it seems to play a role in centrosome maturation, microtubule organization, histone phosphorylation and apoptosis [13].

**Figure 1:**
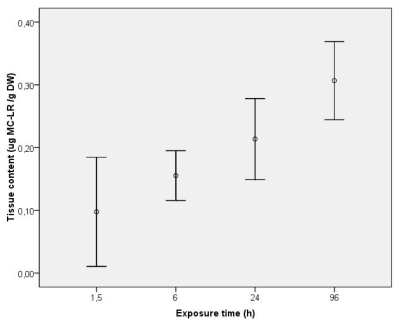
Tissue content (μg MC-LR g^−1^ DW) of unbound MC-LR in the visceral mass during exposure of *C. fluminea* to 5 μg L^−1^ MC-LR for 96 h. Values represent average of three replicates and bars represent confidence interval for mean level (95%).

After designing the specific primers for each of the studied genes, gene expression of PPP1, 2 and 4 was evaluated in the visceral mass of *C. fluminea* in an exposure assay using purified MC-LR. MeOH extractable toxin concentration (unbound MC) was quantified in exposed clam tissues collected during a continuous exposure to 5 μg L^−1^ of purified MC-LR for 4 days (Figure 1). No free MC were detected in control clams after the intoxication period. In the exposure groups, unbound MC-LR was detected in the clams already after 1.5 h, revealing an immediate uptake of the toxin which was continuous, reaching a mean value of 0.213 μg g^−1^ DW at 24 h. The mean maximum uptake detectable level was 0.306 μg g^−1^ DW after 96 h, which reveals an increase of 30% of the accumulated toxin from the end of the first day to the fourth.

**Figure 2:**
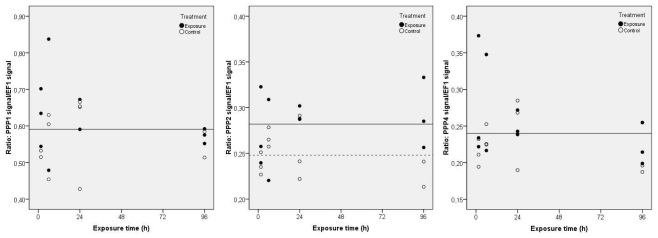
Projection of the normalized gene expression values of PPP1, PPP2 and PPP4 in *C. fluminea* visceral mass after exposure to 5 μg L^−1^ of MC-LR during 96 h, in relation to the studied independent variables, according to the multiple linear regression analysis (Control groups-dashed line; Exposure groups-continuous line). The regression models describing the functions represented in the figure include only the significant regression variables (PPP1: none; PPP2: Treatment; PPP4: none).

During MC-LR exposure, no significant variations were detected for PPP1 and PPP4 transcripts (Figure 2) (same horizontal regression line for controls and exposure groups). However, the regression statistics show significant correlations (*p* < 0.05) in PPP2 gene expression between treatments (Figure 2). A total of 51.4% of the variation in the PPP2 gene expression in clams may be explained by the significant variable (Treatment) (*p* < 0.05) in the multiple linear regression analysis (*F* = 7.175; *p* = 0.014). As a result, PPP2 gene expression is significantly higher in exposed clams during the 96 h, evidencing the need of the clams to repair cells injuries by protein synthesis. There is no significant variation of results over time regardless the applied treatment, which means that the difference between treatments is constant.

**Figure 3:**
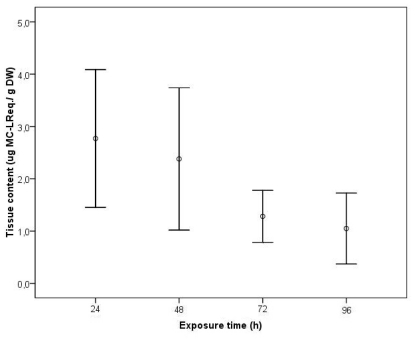
Tissue content (μg MC-LReq. g^−1^ DW) of unbound MC-LReq. in the visceral mass during exposure of *C. fluminea* to 1 × 10^5^ cells cm^−3^ of a *M. aeruginosa* toxic strain for 96 h. Values represent average of three replicates and bars represent confidence interval for mean level (95%).

Based on these last results, we studied alterations in transcriptional patterns in parallel with enzymatic activity of *C. fluminea* for PPP2, induced by a cyanobacteria toxic strain. The unbound MC-LReq. tissue concentration was quantified in exposed clams collected during a continuous exposure to a density of 10^5^ cells cm^−3^ of a *M. aeruginosa* toxic strain for 4 days (Figure 3). No free MC were detected in control clams after the intoxication period and thus no false positives were detected with ELISA. Unbound MC-LReq. was detected in the clams already in day 1, revealing an immediate uptake of the toxin. In the following days, a decrease of the accumulated free MC-LReq. was verified, particularly between days 2 and 3, with a decrease of nearly 55% of the accumulated toxin. The mean maximum uptake detectable level was 2.77 μg g^−1^ DW in the first day. At day 4, the clams retained a concentration 62% lower than the mean maximum uptake level.

**Figure 4:**
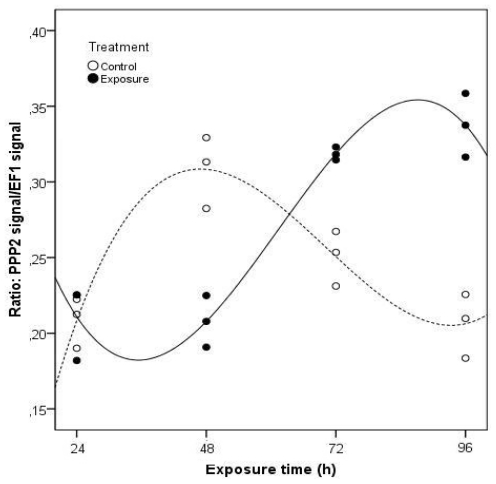
Projection of the normalized gene expression values of PPP2 in *C. fluminea* visceral mass after exposure to 1 × 10^5^ cells cm^−3^ of a *M. aeruginosa* toxic strain during 96 h, in relation to the studied independent variables, according to the multiple linear regression analysis (Control groups-dashed line; Exposure groups-continuous line). The regression models describing the functions represented in the figure include only the significant regression variables (Treatment, Exposure time, Exposure time *squared*, Treatment × Exposure time, Treatment × Exposure time *squared*, Treatment × Exposure time *cubed*).

The clams exposed to the toxic cells of *M. aeruginosa* showed a different pattern of PPP2 gene expression when compared to pure MC-LR exposure (Figure 4). A total of 95.8% of the variation in the PPP2 gene expression in clams may be explained by the significant variables (time of exposure and treatment) (*p* < 0.05) in the multiple linear regression analysis (*F* = 31.240; *p* = 0.000). As a result of these two factors acting either together or independently, the PPP2 mRNA levels are lower for exposed clams in relation to non-exposed clams, between days one and two, contrary to the last two days. After 96 h, PPP2 mRNA expression levels were 61% higher compared to non-exposed clams. As with purified MC-LR, exposure to *Microcystis* cells also induced an enhancement of PPP2 mRNA levels, evidencing as well the need of the clams to repair cells injuries through an increase in protein synthesis in both experiments.

**Figure 5:**
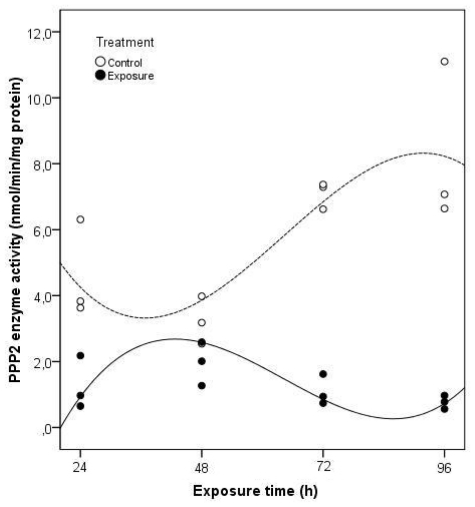
Projection of enzyme activity values of PPP2 in *C. fluminea* visceral mass after exposure to 1 × 10^5^ cells mL^−1^ of a *M. aeruginosa* toxic strain during 96 h, in relation to the studied independent variables, according to the multiple linear regression analysis (Control groups-dashed line; Exposure groups-continuous line). The regression models describing the functions represented in the figure include only the significant regression variables (Treatment, Exposure time, Treatment × Exposure time, Treatment × Exposure time *squared*, Treatment × Exposure time *cubed*).

The four-day exposure to *Microcystis* cells was sufficient to produce a reduction in PPP2 enzyme activity present in clams visceral mass (Figure 5). Taking into account all the significant variables (time of exposure and treatment) (*p* < 0.05) of the multiple linear regression analysis it is possible to explain 93.6% of the total variation in PPP2 enzyme activity in *C. fluminea* (*F* = 25.324; *p* = 0.000). As a result of these two factors, acting either together or independently, the PPP2 enzyme activity in exposed clams is significantly lower during the 96 h of exposure to *M. aeruginosa* toxic strain. This reduction was especially severe after 96 h, with a lower 91% activity compared to non-exposed clams. This kind of response might be related to a disruption in the signaling pathway leading to protein structural damage.

The results from several field studies demonstrate that bivalves accumulate toxins at sites where toxic cyanobacterial blooms occurred [30,31]. Cyanotoxins accumulation in bivalves can result from ingestion of toxic cyanobacteria or exposure to dissolved toxins released to the water after cells lysis. Generally, healthy bloom populations produce low amounts of extracellular toxin, with values ranging from 0.1 to 10 μg L^−1^ [32]. However, concentrations of dissolved toxins in the water column are much higher in ageing or collapsing blooms [32]. One of the most frequently found cyanobacterial toxins in blooms from fresh and brackish waters are the hepatotoxins MC.

This study investigated the *in vivo* effects of MC on gene expression of several PPP in the freshwater clam *C. fluminea* after exposure to dissolved MC-LR and to toxic *M. aeruginosa* cells. We observed a difference in cumulative uptake values and patterns amongst the two different exposure scenarios. Our uptake values are limited to free MC and probably also to MC conjugated with glutathione and cysteine [33]. Since MC covalently bind to PP, a significant part of the toxin becomes undetected by the common methods such as ELISA. Conventional methods such as organic extraction only extract the unbound portions of the toxin [34]. Less unbound MC were detected during the dissolved MC-LR exposure compared to cyanobacterial ingestion. After being exposed for 4 days to 5 μg L^−1^ MC-LR, the clams accumulated a maximum detectable level of 0.306 μg g^−1^ DW in the fourth day. The accumulation of free MC promoted by the exposure to whole *M. aeruginosa* toxic strain cells (4.5 μg MC-LReq. L^−1^) comprehended values between 2.77 and 1.05 μg g^−1^ DW in the first and fourth days, respectively. Although with higher values, the accumulation of unbound MC promoted by the exposure of the clams to cyanobacterial cells are rather not so significant when compared to results of other laboratory experiments. Amorim and Vasconcelos [26] found MeOH extractable MC-LReq. levels in *Mytilus galloprovincialis* (whole body) of 10.7 μg g^−1^ DW, after four days of exposure to a *M. aeruginosa* toxic strain. In a previous work, Vasconcelos [35] found MeOH extractable MC-LReq. levels in the visceral mass of the same marine bivalve of 27.6 μg g^−1^ DW, after sixteen days of exposure, also to a *M. aeruginosa* toxic strain. With *Corbicula* this is the first laboratory study that addresses MC uptake. The lack of data also exists in what it respects to pure MC uptake by bivalves. In one of the few records, the freshwater bivalve *Anodonta grandis simpsoniana* did not accumulate MC after exposure to dissolved MC-LR, during three days [36]. In this study, the measurement of MC in the clams exposed to cyanobacterial cells can be overestimated since much of the toxin detected can arise from intact cyanobacteria in the alimentary tract. *C. fluminea* seems to react to the contact with *M. aeruginosa* cells with MC by closing their valves so the uptake is limited. After the initial ingestion of cells it seems that the clam decreases their filtering rates, avoiding the toxin producing cells. Studies with gastropods with the same two intoxication routes and taking in account emptiness of their gut contents showed differences in MC accumulation, *i.e.*, 1300 times more important in *L. stagnalis* after ingestion of toxic cyanobacteria than after dissolved MC-LR exposure [21,37]. These differences in MC accumulation between both scenarios were also found in our study, which may influence the degree of the subsequent pathological effects.

Nevertheless the low uptake values, both intoxication routes promoted an effect in gene expression of PPP2, which constitutes, according to the *in vitro* studies in mammals [13], one of the most sensitive PPP to MC. The low uptake values associated to a lower sensitivity to MC [13] might also be the reasons for the no observed significant changes in PPP1 and 4 gene expressions upon exposure to dissolved MC-LR. For PPP2, gene expression variations during the 4 days of exposure seem to largely depend on MC uptake and accumulation patterns found in our study for the two routes and forms of exposure. During the dissolved MC-LR exposure, PPP2 expression increased remaining relatively constant over time and concomitant to a relatively constant uptake of the toxin between sampling endpoints. In contrast, upon cyanobacterial exposure, PPP2 transcriptional expression levels in the first two days are lower when compared to non-exposed clams, contrary to the last two days. In this case, intracellular MC were immediately incorporated by *C. fluminea*, but also instantly released. Previous investigations observed as well an immediate uptake and rapid release of MC-LR for *D. polymorpha* and *M. galloprovincialis*, although only when mussels were transferred to toxin-free medium [26,38]. The constant decrease of the MC uptake values since day one found in our study can possibly be explained by a filtration reduction activity by the clams, as a result of the closure of their valves [39]. Interestingly, the major increase above control levels of PPP2 expression after 72 and 96 h to *M. aeruginosa* exposure corresponds to the lower amounts of unbound MC detected during the 4-day exposure, suggesting that most of the accumulated MC at that time is covalently bound. Lance *et al*. [40] found that 17.7 to 66.7% of the total accumulated MC were found in snails exposed to MC producing toxic strain. Both exposure pathways lead to an enhancement of PPP2 gene expression in the visceral mass of *C. fluminea*, although with different patterns. Supporting these results, Huang *et al*. [41] showed that after 7 days of exposure to a cyanobacteria extract (MC-LR, -RR) the expression level of PPP2 in mice liver has increased significantly. For cyanobacterial cells exposure also, the data indicate an increased expression of the PPP2, whereas its activity remains blocked. Several studies show that mRNA levels correlate closely with enzyme activity levels [18,27]. Gehringer *et al*. [18] suggest that the increase in activity was the result of *de novo* synthesis, accompanied by an increase in transcription. In bivalves, as in mammals, the inhibition of PPP activity by MC is also a possible relevant phenomenon which can lead to an imbalance phosphorylation state of several key proteins in many cellular pathways. In this study, toxic *M. aeruginosa* cells producing mostly MC-LR induced a decrease of PPP2 activity in *C. fluminea*. Malbrouck *et al*. [42] also reported a decrease in hepatic PPP activity after injecting intraperitoneally the goldfish *Carassius auratus* with MC-LR.

The major increase above control levels of PPP2 expression detected for both exposure scenarios in this study indicates an enhanced demand for new synthesis of this enzyme. This demand for new synthesis of PPP2 possibly evidences the need of the clams to repair cells injuries. Previously, we showed that a *M. aeruginosa* toxic strain induced changes in protein expression in the freshwater clam *C. fluminea* [43]. We have suggested that the almost exclusive identification of cytoskeletal proteins could reflect PPP2 phosphatase inhibition as major role of MC-related toxicity in bivalves [43]. In this study, protein structural damage may be a consequence of the observed emerging reduction in PPP2 enzyme activity since the first day of exposure to the toxic strain.

## 3. Experimental Section

### 3.1. Test Species and Cultures

For laboratory studies *C. fluminea* with a size of 25–30 mm were manually collected in Rio Minho estuary (Valença, Portugal). At the time, there was no record of cyanobacterial blooms in the area. Animals were acclimated to laboratory conditions in dechlorinated tap water for one month, prior to exposure experiments. Six hundred bivalves were held in two 60 L storage tanks with aerated water at 18 ± 1 °C. The water was exchanged thrice a week. The animals were fed with an algal suspension (*Chlorella vulgaris*, 1 × 10^5^ cells mL^−1^) twice a week. *C. vulgaris* and *M. aeruginosa* strain IZANCYA 2 were cultured in 6 L flasks containing 4 L of Z8 medium [44], using cool white fluorescent light (10 μmol m^−2^ s^−1^) with a light-dark period of 14–10 h, and a temperature of 25 ± 1 °C. IZANCYA 2 produces MC-LR and low amounts of MC-LA and [d-Asp3]-MC-LR [35]. IZANCYA 2 was regularly analyzed to ensure toxin production characteristics.

### 3.2. MC Purification

#### 3.2.1. MC-LR Extraction for Analytical HPLC

Subsamples of *M. aeruginosa* biomass (60–100 mg) were extracted in aqueous methanol (MeOH, HPLC grade) (50%), sonicated in ice bath (60 Hz, 5 min) and the mixture centrifuged (4995 g, 10 min, 4 °C). The supernatant was filtered on GFC 1.2 μm and evaporated in rotavapor until complete dryness. The residue was suspended in aqueous MeOH (50%), filtered on MILLEX^®^GP 0.22 μm then analyzed by HPLC-PDA.

#### 3.2.2. MC-LR Extraction for Semi-Preparative HPLC

Subsamples of *M. aeruginosa* biomass (300–500 mg) were extracted in aqueous MeOH (75%), sonicated in ice bath (60 Hz, 5 min) and the mixture centrifuged (4995 g, 20 min, 4 °C). The supernatant was filtered on GFC 1.2 μm and evaporated in rotavapor until completely dryness. The residue was suspended in aqueous MeOH (50%), filtered on MILLEX^®^GP 0.22 μm then analyzed by HPLC-PDA.

#### 3.2.3. Solid Phase Extraction

The supernatant was transferred to a rounded bottom glass flask (250 mL) and evaporated at 35 °C to about 20 mL using a rotaporator, then applied directly to a Waters Sep-Pak^®^ Vac 6 mL C18–500 mg cartridge, which had been preconditioned by 100% MeOH and distilled water. The column was first washed with 20% MeOH and the toxin then eluted with the 80% MeOH. The MC-containing fraction was evaporated until removing the entire methanol portion. This solution was injected into the HPLC-PDA for analysis.

#### 3.2.4. Quantification of MC-LR

The analytical and semi-preparative analyses of MC-LR were determined according to a modified version of the method applied in Ramanan *et al*. [45]. The chromatographic system consisted of a Waters Alliance e2695 high-pressure liquid chromatograph equipped with a photodiode array detector 2998. The MC-LR analytical assay was performed using a reversed phase column (Merck Lichrospher RP-18 endcapped (25 cm × 4.6 mm, 5 μm)) equipped with a guard column (Merck Lichrospher RP-18 endcapped (4 × 4 mm, 5 μm)) both were kept at 40 °C. The gradient elution utilized MeOH and water both acidified with 0.1% trifluoroacetic acid (TFA, 99.5%) with a flow rate of 0.9 mL/min. The injected volume was 20 μL. The PDA range was 210–400 nm, with a fixed wavelength at 238 nm. The method linearity (*y* = 1.45 × 10^15^ − 1.74 × 10^2^, *R*^2^ = 0.995) was achieved between 0.13 and 10 ppm; limit of detection of the instrument was 0.1 ppm (S/R 3). Sets of 6 samples (in duplicate) were analyzed and correspondent concentrations of MC-LR were obtained by a daily two point calibration method. The MC-LR semi-preparative assay was performed to purify MC-LR for the exposure experiments using a reversed phase column (Phenomenex Luna RP-18 (25 cm × 10 mm, 10 μm)) kept at 30 °C. The gradient elution utilized MeOH and water both acidified with 0.1% TFA with a flow rate of 2.5 mL/min. The injected volume was 500 μL. The PDA range was 210–400 nm, with a fixed wavelength at 238 nm. Peak purity and percentage of MC-LR purified was calculated at 214 nm and 238 nm. Fractions were collected and analyzed by analytical HPLC-PDA.

### 3.3. Exposure Experiments

#### 3.3.1. With Purified MC-LR

Each replicate consisted of five clams exposed in 400 mL of aerated dechlorinated tap water. Both control and treatment were carried out in triplicate. The non-control clams were exposed to 5 μg L^−1^ of purified MC-LR mixed with *C. vulgaris* (1 × 10^4^ cells mL^−1^) to prevent the inhibitory effect by MC alone. Tissues and medium sampling were done after 1.5, 6, 24 and 96 h of exposure to the toxin. The exposure medium was daily renewed. Visceral mass of the collected clams were manually dissected at 4 °C, weighted, frozen with liquid nitrogen and stored at −80 °C until posterior use.

#### 3.3.2. With *M. aeruginosa* Toxic Strain Cells

Exposure experiments were carried out in a continuous flow-through system. In this system, 36 L aquariums containing sand and aerated dechlorinated tap water were used, with 48 clams each. Both control and treatment were carried out in triplicate. Two peristaltic pumps (Gilson Minipuls 3) generated a constant test solution flow of 1500 mL/h. In this way, the aqueous test solution was renewed daily in each aquarium. In the accumulation period, clams were exposed to a constant flow of 25 × 10^5^ cells mL^−1^ h^−1^ of the *M. aeruginosa* strain IZANCYA 2, making a density of 10^5^ cells mL^−1^ (4.5 μg MC-LReq. L^−1^) during 4 days. Control group was fed with *C. vulgaris*. Daily, during these 4 days of the accumulation experiment, 6 animals were randomly collected from each aquarium for toxin analysis, 3 for gene expression and other 3 for enzymatic activity. Visceral mass of the collected clams were manually dissected at 4 °C, weighted, frozen with liquid nitrogen and stored at −80 °C until posterior use.

### 3.4. Toxin Analysis in the Clams

In both exposure experiments (pure or intracellular MC-LR), MC quantification was done in the visceral mass of the bivalves. In the visceral mass, two main areas can be distinguished [46]. The digestive tract which comprises various sections including a large dorsal pouch-shaped stomach and a heavily folded intestinal tract showing numerous loops embedded in the digestive gland and the gonadal tissue [46]. Besides the digestive tract, visceral mass is occupied by the hermaphrodite gonad and other internal organs [46]. Extraction of the MC was done using a modified method described in Amorim and Vasconcelos [26]. *Corbicula* tissues were homogenized in liquid nitrogen until completely disrupted. MC were extracted in 80% methanol (5 mL for 1 g dry weight, 10 °C) with ultrasonication on ice (120 s, 60 Hz). After 24 h, the supernatant was collected and the pellets reextracted with the same procedure. After the 2-day extraction was finished, the two pooled supernatants were combined and centrifuged at 4000 rpm for 30 min. The extracts were diluted (4×) with sterile milliQ water and loaded in a Bond Elut C18 cartridge after being activated with methanol. The cartridge was then rinsed with in sterile Milli-Q water and methanol 20%. Finally, the toxic fraction was recovered with 100% methanol. This solution was completely evaporated and the residues resuspended in Milli-Q water for toxin analysis. For toxin quantification an enzyme-linked immunosorbent assay (ELISA) was used (EnviroGard MCs Plate Kit-Hampshire, UK) with a detection limit of 0.1 ng MC/mL. This assay was already performed using this type of matrix [26]. MC contents in visceral mass of the clams are expressed in μg g^−1^ DW (dry weight). The values were calculated taking into account an extraction recovery of 75%. This value was defined in a previous study in our laboratory using the same procedure for recovery of the toxins [47].

### 3.5. Gene Expression

#### 3.5.1. RNA Extraction and cDNA Synthesis

RNA isolation was performed in the visceral mass of the bivalves exposed to dissolved MC-LR and to *M. aeruginosa* toxic strain cells. RNA isolation from *C. fluminea* tissues (20 mg) was performed according to the manufacturer’s instructions using RNeasy mini Kit (Qiagen, Hilden, Germany), with an additional step of on-column DNA digestion using RNase-Free DNase Set (Qiagen, Hilden, Germany). cDNA synthesis was initiated by incubating 1 μg total RNA with 1 μL Oligo (dT)18 primer mix (Bioline, London, UK) and 1 μL deoxynucleotide triphosphates (dNTPs, 10 mM) (Bioline, London, UK) for 10 min at 65 °C. Reverse Trancription was performed using 4 μL 5× RT buffer (Bioline, London, UK), 1 μL RNase inhibitor (10 U/μL) (Bioline, London, UK) and 0.25 μL MMLV reverse transcriptase (200 U/μL) (Bioline, London, UK). The reaction mixture was incubated at 42 °C for 30 min. The reaction was terminated with an incubation step of 15 min at 70 °C.

#### 3.5.2. Primers Design

All primers were obtained from Invitrogen (Carlsbard, USA). For PPP1, degenerate primers were chosen in the PPP1 conserved coding region of several partial sequences of *Lottia gigantea* built from available EST (Expressed Sequence Tag) records. For PPP2, degenerate primers were chosen in the PPP*2* conserved coding region of the five following mollusks: *Mytilus californianus*, *Crassostrea gigas*, *Crassostrea virginia*, *Chlamys farreri* and *Dreissena polymorpha* (GenBank accession numbers: GE753752; AM856000.1; EH64803; DT717746; AF508223). For PPP4, degenerate primers were chosen in the PPP4 conserved coding region of the four following mollusks: *Crassostrea gigas*, *Crassostrea virginia*, *Chlamys farreri*, *Venerupis philippinarum* (GenBank accession numbers: AM854071.1, CD648951.1, DT717768.1, AM872270.1). The same method was applied to amplify others PPP member (PPP3, PPP5, PPP6) although not successfully due to non-specific amplifications. Specific primers were designed according to *C. fluminea* PPP1, 2 and 4 obtained sequences (Table 1). Specific primers were also designed for elongation factor 1-α (EF1-α) after obtaining *C. fluminea* EF1-α sequence using specific primers designed for the freshwater mussel *D. polymorpha* [48]. The PCR products using the specific primers were sent for sequencing to confirm the specificity of the amplified products.

#### 3.5.3. Quantitative RT-PCR

Quantitative RT-PCR was performed using a iCycler iQ™ Real-Time PCR Detection System (Bio-Rad). Specific primers were used to amplify cDNA of PPP1, PPP2 and PPP4. EF1-α cDNA was amplified as a control. EF1-α was used as a control gene for DNA level normalization. The EF1-α was previously used as housekeeping gene in other studies [48,49] and accordingly we observed in the experiment an absence of changes in the expression levels after the treatment. Each reaction mixture consisted of 4 μL cDNA template, 0.25 μM of each primer, 1× IQ SYBR Green Supermix (Bio-Rad) and water to adjust to 20 μL final reaction volume. Reaction mixtures were subjected to the following cycling conditions: 5 min at 95 °C to denature DNA and activate Taq polymerase; 40 cycles of 15 s at 95 °C, 30 s at 58 °C and 30 s at 72 °C. All reactions were run on duplicate in a 96-well plate, including a negative control for each set of primers. Melting curves (81 steps of 30 s, from 55.0 to 95.5 °C, with a temperature gradient increase of 0.5 °C per step) were generated for PCR products to confirm the specificity of the assays and to check the occurrence of primer dimers. A dilution series was also prepared to test the efficiency of PCR amplifications. The PCR efficiency for each gene amplification varied from 96.6% to 110.2%. A standard curve for qPCR was determined as a correlation between gene copy numbers and the cycle threshold (*CT*). For each gene, standards were prepared with the primers used in the Q-PCR to amplify the cDNA in a normal PCR reaction. The purified PCR products were used as standard samples, and 10-fold serial dilutions of these samples were analyzed by qPCR. Gene copy numbers of the standard samples were determined using the following formula: copy number = [DNA quantity (g) × Avogadro constant (6.022 × 10^23^ copies mol^−1^)]/[DNA length (bp) × 660 (g/mol/bp)] [50].

### 3.6. Enzyme Analysis

PPP2 activity was performed in the visceral mass of the bivalves exposed to *M. aeruginosa* toxic strain cells according to the manufacturer’s instructions, using Serine/Threonine Phosphatase Assay System Kit (Promega Corporation, Madison, WI). This method determines the amount of free phosphate generated in a reaction by measuring the absorbance of molybdate malachite green: phosphate complex using the synthetic phosphopeptide RRA(pT)VA as substrate. Protein content of the samples was quantified according to Bradford [51].

### 3.7. Statistical Analysis

Pearson’s coefficient (*R* Pearson) was calculated to assess the existence of a correlation between the fresh and dry weights of clams (*n* = 30).

PPP gene expression and enzyme activity responses (dependent variables) were examined with saturated orthogonal multiple linear regression analysis and subsequent variance analysis (ANOVA) [52,53]. The regression model was defined as *y* (dependent variable) = *b_0_* + *b*_1_ × Treatment + *b*_2_ × Exposure time + *b*_3_ × Exposure time *squared* + *b*_4_ × Exposure time *cubed* + *b*_5_ × Treatment × Exposure time + *b*_6_ × Treatment × Exposure time *squared* + *b*_7_ × Treatment × Exposure time *cubed*. The independent variable Treatment is composed of two modalities or conditions: control group and group of organisms exposed to MC. The independent variable Exposure time is composed of four modalities or times of exposure: 1.5, 6, 24 and 96 h for MC-LR exposure and 24, 48, 72 and 96 h for cyanobacterial cells exposure. The normality of each distribution was verified by Shapiro-Wilk test and variance homogeneity by Levene’s Test of Equality of Error Variances. A significant level (α) of 0.05 was used for the multiple regression analysis, for normality and variance homogeneity tests. All graphics and statistical analysis were carried out using SPSS 11.0 for Windows (Chicago, IL, USA).

## 4. Conclusions

This paper is the first to sequence PPP1, PPP2 and PPP4 in *C. fluminea*. The study showed that despite low uptake levels, the exposure of *C. fluminea* to an environmentally relevant concentration of purified MC-LR resulted in a significant induction of PPP2 gene expression in the visceral mass. In contrast, MC-LR did not cause any significant changes on PPP1 and PPP4. The exposure of *C. fluminea* to a strain of *M. aeruginosa* producing mostly MC-LR also caused *in vivo* induction of PPP2 on the transcript level preceded by an immediate reduction of PPP2 enzyme activity.

Considering all the results we can say that the exposure to an ecologically relevant concentration of pure or intracellular MC (-LR) promoted an *in vivo* effect on PPP2 gene expression in *C. fluminea*. The PPP2 expression variations during the four days of exposure seem to largely depend on MC accumulation patterns found in our study for the two routes and forms of exposure.

Apart from the ecological relevance, this study itself may also contribute to the PPP research. Having a major role on MC-related toxicity, further *in vivo* studies should continue to study how the PP activity and gene expression are affected raising the knowledge about the molecular mechanisms of MC induced toxicity in bivalves.

## Figures and Tables

**Table 1:** Sequences of primers used for RT-PCR.

	Sequence of Foward primer (5′–3′)	Sequence of Reverse primer (5′–3′)	Amplified fragment size (bp)
**PPP1**	AATGTGCCAGCATCAACAGA	ATCTGTTGGCCGCATAATTC	206
**PPP2**	ACGGCAATGCTAATGTTTGG	GACCCTCATGTGGAACCTCT	171
**PPP4**	ACGAGGGAATCATGAAAGCCGTC	TCGCGGACTCACTCCCCATCC	301
**EF1-α**	CGTTGGTGTCAACAAGATGG	TACAGCCCAACCCTTGTACC	202
